# Phase I and phase II sonidegib and vismodegib clinical trials for the treatment of paediatric and adult MB patients: a systemic review and meta-analysis

**DOI:** 10.1186/s40478-019-0773-8

**Published:** 2019-07-30

**Authors:** Yuchen Li, Qingkun Song, Bryan W. Day

**Affiliations:** 1 Department of Cell and Molecular Biology, QIMR Berghofer Medical Research Institute, Brisbane, 4006 Australia; 2 Faculty of Medicine, the University of Queensland, Brisbane, 4006 Australia; 3 Department of Science and Technology, Beijing Shijitan Hospital, Capital Medical University, Beijing, 10038 China; 4 Department of Clinical Epidemiology and Evidence-based Medicine, Beijing Shijitan Hospital, Capital Medical University, Beijing, 10038 China; 5 School of Biomedical Sciences, Faculty of Health, Queensland University of Technology, Brisbane, 4059 Australia; 6 School of Biomedical Sciences, The University of Queensland, Brisbane, 4072 Australia; 7 Sid Faithfull Brain Cancer Laboratory, Brisbane, 4006 Australia

**Keywords:** Medulloblastoma, Sonic hedgehog pathway, SMO inhibitor, Sonidegib, And vismodegib

## Abstract

**Background:**

Medulloblastoma (MB) is the most common malignant brain tumour in children but also rarely occur in adults. Sonic Hedgehog (SHH) driven MB is associated with aberrant activation of the SHH signalling pathway. SMO inhibitors, sonidegib and vismodegib, have been used as selective antagonist of the hedgehog pathway that acts by binding to SMO, and inhibits activation of the downstream hedgehog target genes. Several clinical trials investigating SMO inhibitors for the treatment of relapsed MB patients have been published.

**Methods:**

We conducted a systemic review and meta-analysis among these Phase I and II clinical trials. The pooled effect of SMO inhibitors in relapsed MB were analysed using Reviewer Manager 5.3 software. The clinical efficacy of SMO inhibitors on SHH subtype of MB were measured by the objective response rate. The risk difference was obtained by comparing the ORR between SHH and non-SHH subtypes of MB.

**Results:**

The five studies all had clear criteria for patient recruitment, adequate follow-up time for endpoint assessment and clear definition of tumour responses. MB patients had good compliance in the trials. The pooled objective response rate (ORR) of SMO inhibitor was 37% and 0 against SHH-driven and other MBs. The pooled ORR of sonidegib was 55% among MB^SHH^ and 0 among MB^non-SHH^ subgroup. Vismodegib also had no efficacy on non-SHH subtype of MB. The sonidegib against SHH-driven MB produced the ORR 1.87-fold higher than that of vismodegib (95%CI 1.23, 6.69). Among paediatric patients, the efficacy of sonidegib was 3.67-fold higher than vismodegib (*p* < 0.05). A total of 320 cases received SMO inhibitor therapy and 36 cases reported grade 3/4 dose-limiting toxicity (DLT). The rate of grade 3/4 DLT was similar between patients receiving vismodegib and sonidegib (11.6% vs. 11.2%).

**Conclusion:**

Sonidegib and vismodegib were well tolerated and demonstrated anti-tumour activity in SHH-driven paediatric and adult MB by effectively inhibiting Hh signalling. These results support the ongoing clinical trials using SMO inhibitors in combination with conventional chemotherapies for the treatment of relapsed MB^SHH^.

## Introduction

Medulloblastoma (MB) is the most frequent malignant brain tumour (WHO grade IV) to occur in children and remains the leading cause of cancer-related mortality in childhood. The peak age of diagnosis is approximately 7 years of age, tumours can also rarely occur during adulthood in some individuals [15]. International consensus recognises four distinct MB molecular subgroups: WNT (MB^WNT^), SHH (MB^SHH^), Group 3 (MB^Grp3^) and Group 4 (MB^Grp4^) [14]. This review will focus largely on the SHH subgroup which accounts for approximately 30% of all MB cases [20]. MB^Grp3^ and MB^Grp4^ have the worst prognosis while MB^WNT^ is the most favourable [20]. MB^SHH^ falls in between, with a 5-year overall survival (OS) rate of approximately 70% [29]. Despite a relatively good prognosis for MB^WNT^ and MB^SHH^ tumours, patients experience severe long-term side effects, and the development of secondary, therapy-induced, malignancies in later life [17, 30]. Therefore, more specific and less toxic therapies are required to treat these tumours. Here, we review the current clinical progress to-date of two novel SMO inhibitors, sonidegib (LDE225) and vismodegib (GDC-0449) for the treatment of MB^SHH^.

Aberrant activation of the Sonic Hedgehog (SHH) signalling pathway has been found in familial and sporadic MB patients [13]. Genetic alterations lead to constitutive activation of the hedgehog pathway in MB [24]. Moreover, overexpression of the hedgehog ligand has been linked with the pathogenesis of a number of sporadic cancers, such as pancreatic, colorectal, prostate, prostate, breast and lung [31]. Inhibition of the hedgehog pathway has been reported by using two novel SMO inhibitors in MB, sonidegib (LDE225) and vismodegib (GDC-0449). Both agents are selective antagonists of the hedgehog pathway that act by binding to SMO, and inhibit activation of downstream hedgehog target genes [9, 12]. Vismodegib has been approved by the U.S. Food and Drug Administration (FDA) for the treatment of metastatic or locally advanced non-resectable basal cell carcinoma (BCC) [26]. A Phase I clinical trials of vismodegib has demonstrated a 60% response rate in locally advanced or metastatic BCC [32]. Furthermore, one case study indicated a transient and incomplete response in a patient with metastatic MB [27]. Current clinical trial data has shown varying responses to the efficacy of SMO inhibitors in relapsed or refractory paediatric and adult MB. We therefore performed a systemic review and meta-analysis of clinical trial cohort data to assess their safety and response rate for the treatment of patients with MB.

## Methods

### Databases

We searched articles from PubMed (https://www.ncbi.nlm.nih.gov/pubmed/), the Cochrane Library (https://www.cochranelibrary.com/search) and the Embase database (https://www-embase-com.ezproxy.library.uq.edu.au/#search) accessed from the University of Queensland library. Clinical trial data with a publication date before May 2019 were included in this review.

### Search terms and strategies

The search terms included medulloblastoma or MB or brain tumour or CNS tumour and SMO or smoothened or vismodegib or sonic hedgehog or sonidegib or SHH. In PubMed, the additional filters were ‘clinical trial’. In the Cochrane Library database, the additional filter was ‘trials’. In Embase database, the filter was ‘randomized controlled trial’.

### Included studies

The study design was defined as a clinical trial, and the excluded designs were prospective studies, reviews, animal studies and other basic science studies. The included studies were either phase I or phase II clinical trials, which had to provide dose-limited toxicity (DLT) and response rates (RR). This study focused on original clinical trials but not the re-analysis of previous data review and comments.

### Data extraction

A double-blind extraction of the data was conducted by two health professionals. The extracted data included the phase of trials, authors, publication year, drug, number of patients, the eligible disease, and daily dose of the drug, tumour responses, dose-limiting toxicity (DLT) and safety. The tumour responses were determined according to the RESIST v1.0 criteria and/ or Neuro-Oncology criteria of tumour response, including complete response (CR), partial response (PR), stable disease (SD) and progressed disease (PD). The outcome events were defined as CR and PR.

### Data synthesis

The pooled effect of SMO inhibitors in relapsed MB were synthesized using Reviewer Manager 5.3 software. The clinical efficacy of SMO inhibitors on SHH subtype of MB were measured by the objective response rate (ORR, CR + PR/all cases). The risk difference was obtained by comparing the ORR between SHH and non-SHH subtypes of MB. The difference of clinical efficacy between vismodegib and sonidegib was estimated by risk ratio with reference of vismodegib. The heterogeneity of pooled effects was indicated by I^2^. The pooled effect was synthesized under the fixed model with the non-significant heterogeneity (*p* > 0.05) or the random model with a significant heterogeneity (*p* < 0.05).

## Results

**Fig. 1:**
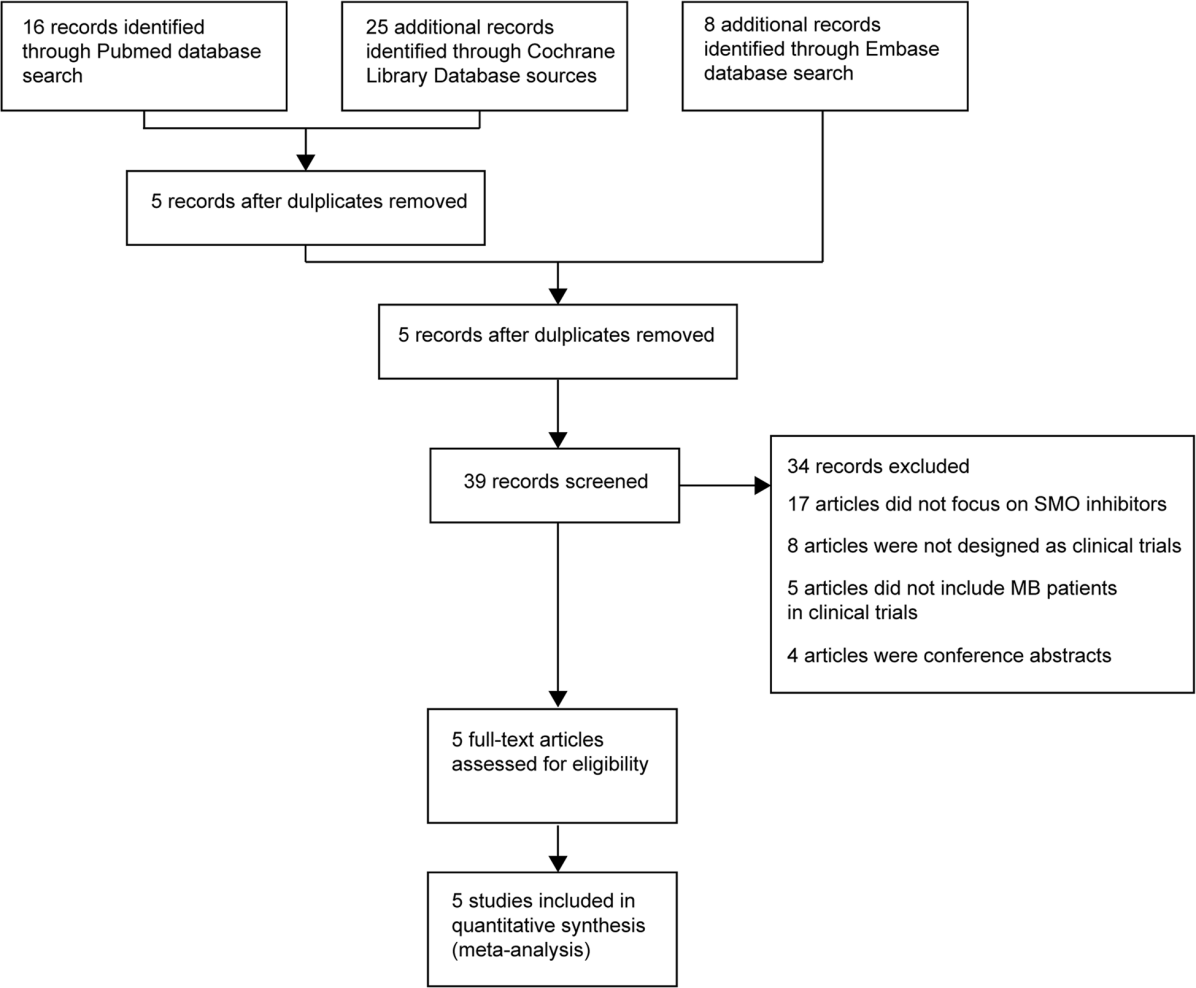
Flow diagram of study search and inclusion

Forty-nine articles were obtained from PubMed, the Cochran Library and Embase database with 10 duplicates removed (Fig. 1). Thirty-four articles were excluded as they were conference abstracts, unrelated to SMO inhibitors, not designed as clinical trial, or did not include MB patients in clinical trials (Fig. 1). Five articles were assessed for eligibility and included into the meta-analysis for safety and response rate evaluation of vismodegib and sonidegib in MB treatment (Fig. 1).

The included trials are composed of four phase I and two phase II trials (Table 1). Among the 320 subjects recruited in the trials, 138 cases were diagnosed as MB (Table 1). The trials recruited relapsed/refractory MB patients and the research endpoints contained safety and tumour responses (Table 1).

**Table 1:** Characteristics of included studies

Trial	Phase	SMO inhibitor	Sample size	No. of MB patients	Disease stage	Endpoints
LoRusso 2011	I	Vismodegib	68	1	Refractory	Safety and tumor responses
Gajjar 2013	I	Vismodegib	33	33	Refractory or relapsed	Safety and tumor responses
Rodon 2014	I	Sonidegib	103	9	Relapsed	Safety and tumor responses
Robinson 2015	II	Vismodegib	40	40	Refractory or recurrent	Safety and tumor responses
Kieran 2017	I and II	Sonidegib	76	55	Phase I: Progressed Phase II: Recurrent or relapsed	Safety and tumor responses

The five studies all had clear criteria for patient recruitment, adequate follow-up time for endpoint assessment and clear definition of tumour responses (Table 2). The MB patients had good compliance in the trials and signed consent for MB subtyping classification (Table 2).

**Table 2:** The quality check based on MB patients in each study

Study	Clear criteria of patient recruitment	Adequate follow-up time for endpoints	Clear definition of tumor responses	Good compliance	MB with SHH
LoRusso 2011	Yes	Yes	No	Yes	Yes
Gajjar 2013	Yes	Yes	Yes	Yes	Yes
Rodon 2014	Yes	Yes	Yes	Yes	Yes
Robinson 2015	Yes	Yes	Yes	Yes	Yes
Kieran 2017	Yes	Yes	Yes	Yes	Yes

**Fig. 2:**
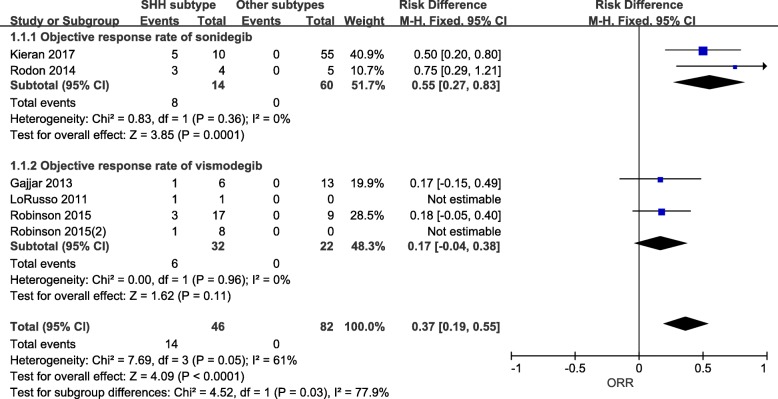
The objective response rate of sonidegib and vismodegib in MB patients. SMO inhibitors in relapsed MB were analysed using Reviewer Manager 5.3 software. No efficacy in non-SHH subtype of MB for either agent was detected. While the pooled ORR of sonidegib and vismodegib was 55 and 17% among MB^SHH^ patients, respectively

There were 14 MB^SHH^ patients and 60 MB^non-SHH^ patients studied for sonidegib and 32 patients and 22 MB^non-SHH^ patients studied for vismodegib (Fig. 2). The pooled ORR of SMO inhibitor was 37% for SHH-driven disease, but zero for other MB subtypes (Fig. 2). The pooled ORR of sonidegib was 55% among MB^SHH^ and 0 among MB^non-SHH^ subgroup (Fig. 2). Vismodegib also had no efficacy on non-SHH subtype of MB. Though vismodegib produced a 17% ORR, the effect size was not significant (Fig. 2). The heterogeneity was not significant between included studies (Fig. 2).

**Fig. 3:**
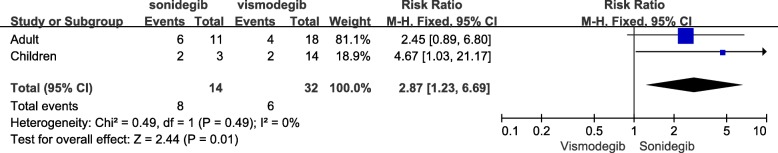
The pooled clinical efficacy of sonidegib and vismodegib in paediatric versus adult SHH-driven MB. Efficacy was analysed using Reviewer Manager 5.3 software. In adult patients, sonidegib had a 1.45-fold higher effect, but the difference was not significant. In contrast, the efficacy of sonidegib was significant showing a 3.67-fold higher effect than vismodegib in paediatric patients (*p* < 0.05)

The sonidegib against SHH-driven MB produced the ORR 1.87-fold higher than that of vismodegib (95%CI 1.23, 6.69, Fig. 3). There were 11 adult MB^SHH^ patients who received sonidegib and 18 adult MB^SHH^ patients who received vismodegib, respectively (Fig. 3). Among adult patients, sonidegib had a 1.45-fold higher effect than vismodegib, but the difference was not significant (Fig. 3). There were 3 paediatric SHH-driven MB patients who received sonidegib and 14 paediatric MB^SHH^ patients who were given vismodegib, respectively (Fig. 3). However, among paediatric patients, the efficacy of sonidegib was 3.67-fold higher than vismodegib (*p* < 0.05, Fig. 3).

A total of 320 cases received SMO inhibitor therapy and 36 cases reported grade 3/4 DLT, including γ-glutamyl transferase, hypokalemia and thrombocytopenia. 16 cases received vismodegib at doses of ≥150 mg/kg reported grade 3/4 DLT. One paediatric patient received sonidegib at doses of 372 mg/kg and the 19 adult patients that received sonidegib ≥800 mg/kg were reported to have grade 3/4 DLT. The rate of grade 3/4 DLT was similar between patients receiving vismodegib and sonidegib (11.6% vs. 11.2%).

## Discussion

The standard of care for MB patients consists of surgical resection followed by craniospinal irradiation and adjuvant chemotherapy, including cyclophosphamide, cisplatin, vincristine, lomustine, etoposide, either alone or in combination [3, 20]. Recently, MB has been further stratified into 12 subtypes demonstrating the extent of heterogeneity that exists within this disease entity. With respect to MB^SHH^, four clinically and cytogenetically distinct groups have been identified: α, β, γ and δ. SHH- α tumours mainly affect children (age 3–16), and are enriched for *MYCN* amplification, GLI2 amplification, and *TP53* mutations, and have the worst prognosis [2, 28]. They also have specific copy-number aberrations (CNAs), such as 9q loss, 10q loss, 17p loss, and YAP1 amplifications [2]. SHH-β and γ are enriched in infant MB patients (age < 3). However, the prognosis of β tumours is worse than γ tumours because of the high frequency of metastasis in SHH-β. Adult SHH is defined as SHH-δ and is enriched for either *PTCH1*, *SMO* or *TERT* promoter mutations, and have a favourable prognosis [2, 10]. Compared to other subgroups, SHH tumours more frequently recur locally in the original resection cavity [18]. The recent WHO classification defined young children and *TP53* wild type patients as low risk and average risk patients [18], while patients with *TP53*-mutated MB^SHH^ have a worse prognosis [18].

Aberrant activation of the SHH signalling pathway has been found in familial and sporadic MB patients [13]. Genetic alterations, including mutations in *PTCH, SUFU,* and *SMO* lead to constitutive activation of the hedgehog pathway in BCC, rhabdomyosarcoma and MB [24]. Moreover, overexpression and/or inappropriate expression of the hedgehog ligand has been linked with the pathogenesis of a number of sporadic cancers, such as pancreatic, colorectal, prostate, breast and lung [31]. Therefore, hedgehog pathway signalling has emerged as a legitimate targetable pathway in a number of cancers including SHH-driven MB.

**Fig. 4:**
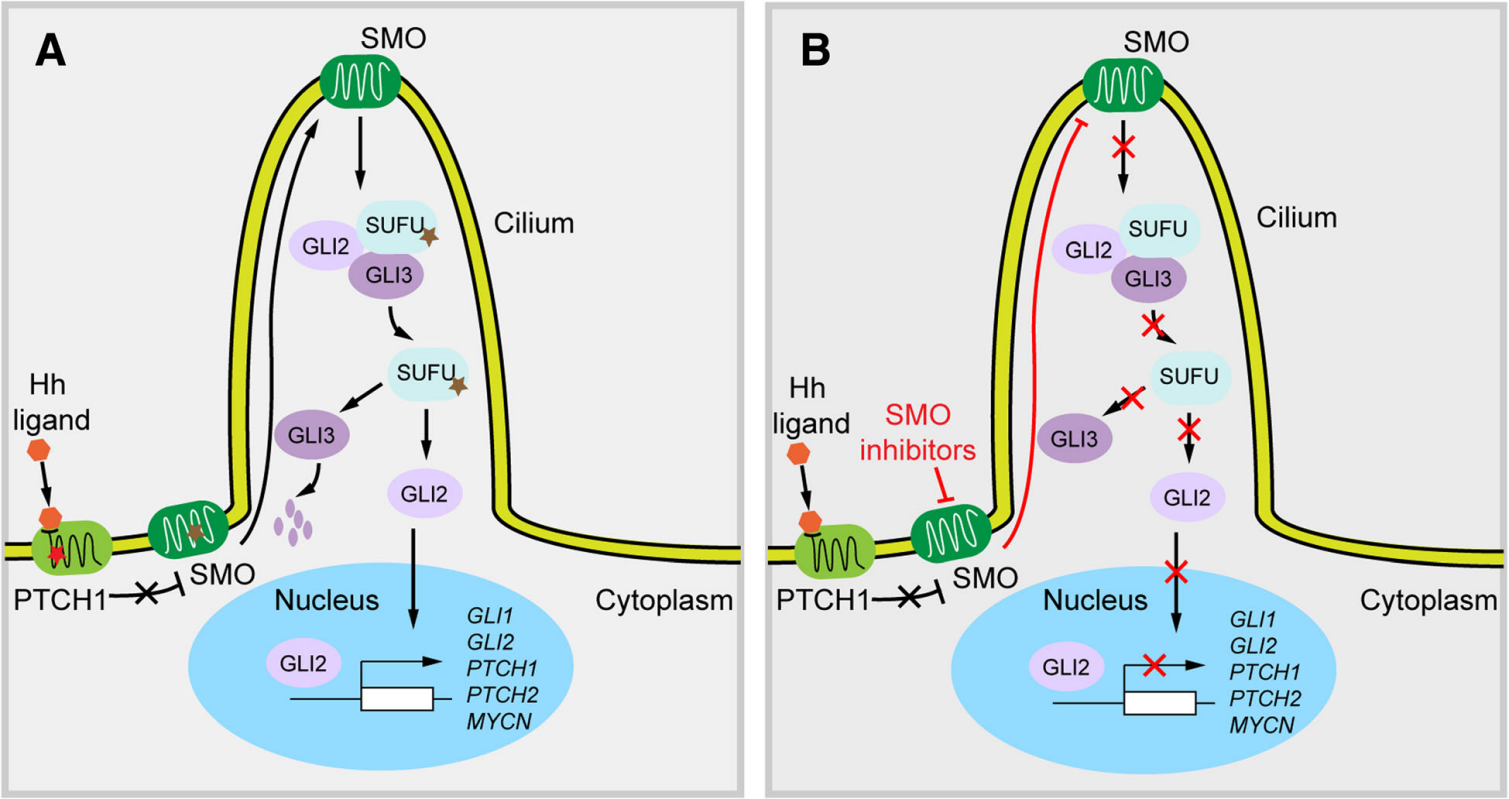
Hedgehog signalling and SMO inhibitors action in MB. **a**. Hedgehog (Hh) proteins (Sonic, Indian, or Desert Hedgehog) bind to PTCH1 transmembrane protein. Binding to PTCH1 relieves inhibition of smoothened (SMO). Active SMO moves to cilium and promotes to release suppressor of fused (SUFU) inhibition of glioma-associated oncogene (GLI) proteins. Activated GLI proteins then translocate to the nucleus to affect transcription of SHH target genes (ie, *GLI1*, *GLI2*, *PTCH1*, *PTCH2*, and *MYCN*). Vismodegib and sonidegib bind to the extracellular domain of SMO, inhibiting downstream signalling. Most commonly mutations in MB associated with Hh pathway includes, mutations in PTCH1 (red star, favourable prognostic mutation), SMO and SUFU (brown star, worse prognostic mutations). **b**. SMO inhibitors inhibit Hh pathway signalling by preventing activation of SMO

In the absence of hedgehog ligand binding, its receptor PTCH inhibits Smoothed (SMO) and acts as a negative regulator of the hedgehog signalling pathway. Hedgehog signalling is activated when the extracellular Hh protein binds to PTCH, preventing its inhibition of SMO (Fig. 4). Activated SMO localises to cilium and initiates a downstream signalling cascade, involving suppressor of fused (SUFU), also activation of glioma-associated oncogene (GLI) transcription factors that translocate to the nucleus and induce hedgehog pathway target gene expression [9]. Both vismodegib and sonidegib bind to SMO, where they act as antagonists, markedly inhibiting downstream activation of Hh pathway signalling, even in the absence of PTCH1. Earlier preclinical studies have shown anti-tumour activity in MB mouse models by using vismodegib [21]. It has also been demonstrated that sonidegib effectively penetrates the blood-brain barrier (BBB) in preclinical studies, making these SMO inhibitors potential candidates for MB treatment [16]. Oral administration of the drug in mouse MB genetic engineered models led to complete inhibition of GLI1 and tumour regression [1]. However, the response to SMO inhibitors were variable in these studies, likely reflecting tumour heterogeneity. They were found ineffective in tumours driven by mutations in SHH pathway genes downstream of SMO, while showed great efficacy in MB^SHH^ driven by mutations upstream of SMO [4, 11, 25].

In the Phase I and Phase II clinical trials discussed in this paper, Hh pathway activation was identified by two methods, either by 5-gene signature RT-PCR assay [9, 12, 24] or immunohistochemistry [5, 23]. Though SMO inhibitors introduced an optimistic response rate to MB, the efficacy of sonidegib was better than vismodegib, especially among paediatric SHH-driven MB patients. However, this conclusion was made based on 3 paediatric patients in the trial. More patients need to be recruited to make a final conclusion.

The pharmacokinetics of vismodegib showed a substantial interpatient variability in all aspects of vismodegib disposition, including variable solubility-limited absorption in the intestine after oral administration, limited metabolic elimination, and interactions with plasma protein alpha-1-acid glycoprotein (AAG) [5, 7]. While sonidegib exposure in children is consistent with that observed in adults for equivalent mg/m^2^ doses [9, 24]. Other possible reasons for why patients have seen variable responses could include mutations associated with Hh signalling pathway. For instance, a mutation in the extracellular domain of SMO, D473H, prevents vismodegib binding [26]. Other resistance mechanisms occurring at the cell surface such as the loss of primary cilia can occur [6, 33]. Cilia is the primary site where activated SMO is trafficked to initiate downstream signalling, cilia loss enables low but constitutive Hh signalling protecting tumour cells from the action of vismodegib or sonidegib [6, 33].

A vismodegib Phase II trial demonstrated a potential benefit of prolonged PFS in SHH-driven MB patients with somatic loss of heterozygosity (LOH) of *PTCH1* compared to MB^non-SHH^ and MB^unknown^ patients [23], suggesting that activity is not limited to objective response. However, SMO inhibitors response variability is based on the position of mutations relative to SMO. Aberrations in *PTCH1* results in favourable outcomes, whereas aberrations in downstream of *SMO*, *GLI2* or *SUFU*, are associated with no response to SMO inhibitors [23]. From DNA methylation and next-generation sequencing data of SHH-driven MB patients, researchers reported that adult MB^SHH^ (SHH-δ) patients will most likely benefit from the SMO inhibitors since they harbour mutations in either *PTCH1* or *SMO* [10]. In contrast, infant (SHH-β and γ) and children (SHH-α) SHH-driven MB frequently have mutations downstream of *SMO* and will unlikely benefit from treatment [10]. Furthermore, MB^SHH^ in children with strong diffuse staining of P53 also respond poorly to SMO inhibitors [23]. Therefore, it is critical to identify MB^SHH^ patients with mutations upstream of *PTCH1* that respond to vismodegib and sonidegib and stratify MB^SHH^ patients for treatment. At present, this testing requires specialist services and is reliant on the availability of quality tissue for analysis.

Irrespective of tumour type, 36 patients were reported experiencing grade 3/4 DLT when receiving SMO inhibitors. Sonidegib and vismodegib are well tolerated and safe in MB patients. All clinical trials demonstrated the safety and feasibility of both drugs in children and adult MB patients. Vismodegib is as effective as sonidegib, but it seems to provoke more severe adverse events including grade 3 muscle spasms and atrial fibrillation [8]. Increased creatine phosphokinase (CPK) elevation was observed in paediatric patients more so than in adults following administration of sonidegib [9], but the underlining reasons were not elaborated. The Phase I/II study of sonidegib also demonstrated permanent bone growth defects in paediatric patients, which were not reported in the clinical trial of vismodegib [9, 23]. Since SHH-driven MB is common in infants and children, the potential risks of using Hh pathway inhibitors should be advised to patients and their families.

There is one registered Phase I clinical trial with sonidegib for the treatment of MB which is currently recruiting (NCT03434262). The trial is being conducted at St. Jude Children’s Research Hospital evaluating sonidegib in combination with ribociclib for the treatment of refractory or recurrent MB^SHH^ patients with 9q loss or a PTCH1 mutation. This study will primarily determine the safety and tolerability of the modalities. Another ongoing trial (NCT01878617) is a Phase II clinical trial of vismodegib in combination with chemotherapy (cisplatin, vincristine, cyclophosphamide) for the treatment of standard and high risk newly diagnosed MB^SHH^ patients. This study will evaluate the feasibility and toxicity of oral maintenance therapy with vismodegib following conventional adjuvant chemotherapy.

Small sample size was the main limitation of this study. The comparisons between clinical efficacy of sonidegib and vismodegib were not adjusted for the confounding factors existing across the studies. The two-arm randomized control trial should be proposed by comparing sonidegib and vismodegib. MB^SHH^ tumours recur mostly in the local tumour bed [19], and the molecular subgroup of the tumour is not significantly altered at recurrence [19]. Currently, there are lack of treatment regimens for relapsed or refractory MB. SMO inhibitors might provide a useful therapeutic option to further extend survival in this treatment refractory group. To avoid and overcome SMO inhibitor resistance, combination therapies will likely be needed. Frequent aberrations in genes involved in phosphoinositide 3-kinase (PI3K) signalling are commonly found in MB^SHH^, therefore the use of a PI3K inhibitor in combination with SMO inhibitor may decrease drug resistance and recurrence [1, 10, 22]. Genome sequencing and complete molecular profiling are needed to further identify patients who will benefit from SMO inhibitors and to study mechanisms of resistance in these patients. In summary, this review highlights that sonidegib and vismodegib were well tolerated and demonstrated anti-tumour activity in SHH-driven MB by effectively inhibiting Hh signalling. These results support the ongoing clinical trials of using SMO inhibitors in combination with conventional chemotherapies for the treatment of relapsed MB^SHH^.

## Abbreviations

AAG: Alpha-1-acid glycoprotein
BBB: Blood brain barrier
BCC: Basal cell carcinoma
CNAs: Copy-number aberrations
CPK: Creatine phosphokinase
CR: Complete response
DLT: Dose-limiting toxicity
GLI: Glioma-associated oncogene
LOH: Loss of heterozygosity
MB: Medulloblastoma
ORR: Objective response rate
OS: Overall survival
PD: Progressed disease
PFS: Progression-free survival
PI3K: Phosphoinositide 3-kinase
PR: Partial response
RR: Response rate
SD: Stable disease
SHH: Sonic hedgehog
SMO: Smoothened
SUFU: Suppressor of fused
